# Ability of the Premise Condition Index to Identify Premises with Adult and Immature *Aedes* Mosquitoes in Kampong Cham, Cambodia

**DOI:** 10.4269/ajtmh.19-0453

**Published:** 2020-04-06

**Authors:** John Hustedt, Dyna Doum, Vanney Keo, Sokha Ly, BunLeng Sam, Vibol Chan, Sebastien Boyer, Marco Liverani, Neal Alexander, John Bradley, Didot Budi Prasetyo, Agus Rachmat, Sergio Lopes, Rithea Leang, Jeffrey Hii

**Affiliations:** 1 Malaria Consortium, Phnom Penh Center, Phnom Penh, Cambodia;; 2 London School of Hygiene and Tropical Medicine, London, United Kingdom;; 3 Cambodian National Dengue Control Program, Phnom Penh, Cambodia;; 4 World Health Organization, Phnom Penh, Cambodia;; 5 Institut Pasteur du Cambodge, Phnom Penh, Cambodia;; 6 US Naval Medical Research Unit-2, Phnom Penh, Cambodia

## Abstract

*Aedes*-transmitted diseases, especially dengue, are increasing throughout the world and the main preventive methods include vector control and the avoidance of mosquito bites. A simple Premise Condition Index (PCI) categorizing shade, house, and yard conditions was previously developed to help prioritize households or geographical areas where resources are limited. However, evidence about the accuracy of the PCI is mixed. The current study aimed to contribute to a better understanding of the relevance by collecting data from 2,400 premises at four time points over 1 year in Kampong Cham, Cambodia. Regression models were then used to identify associations between PCI and *Aedes* adult female mosquitoes and pupae. In addition, receiver operating characteristic curves were used to measure the ability of PCI to identify premises in the top quartile of mosquito abundance. The density of adult *Aedes* females was positively associated with PCI at the household (ratio of means = 1.16 per point on the PCI scale) and cluster level (ratio of means = 1.54). However, the number of *Aedes* pupae was negatively associated with PCI at the household level (rate ratio = 0.74) and did not have a statistically significant association at the cluster level. Receiver operating characteristic curves suggest the PCI score had “rather low accuracy” (area under the ROC curve = 0.52 and 0.54) at identifying top-quartile premises in terms of adult female *Aedes* and pupae, respectively. These results suggest that caution is warranted in the programmatic use of PCI in areas of similar geography and mosquito abundance.

## INTRODUCTION

Dengue is the most rapidly spreading mosquito-borne viral disease in the world and is caused by bites of infected *Aedes* mosquitoes, principally *Aedes aegypti*.^1^ Dengue is endemic worldwide, with a high concentration in the Asian region, which shoulders 70% of the global disease burden. Although a number of promising vaccine candidates are in preclinical and clinical development,^2^ methods of genetic control of mosquitoes are being developed,^3,4^ and Wolbachia-infected mosquitoes show promise,^5,6^ these interventions are unlikely to eliminate dengue on their own.^7^ Therefore, vector control will remain a key component of dengue control in the short and medium term.

One important aspect of vector control is the elimination of the most productive breeding sites.^8^ For example, one study in Australia found that one well and one rainwater tank were responsible for 28% of all immature larvae out of 1,349 premises inspected.^9^ Similarly, in Cambodia, large water jars, drums, and concrete tanks were found to harbor 90% of the pupal biomass.^10^ In addition, studies documented that particularly high levels of *Aedes* productivity can be found in “key premises,”^11–14^ defined as those with three or more positive containers.^9^ In Australia, 1.9% of premises accounted for 47.2% of positive containers.^9^ In Ecuador, 11% of households contained 81.7% of pupae during the rainy season and 5% of households contained 80% of pupae during the dry season.^12^ Thus, it is clear that the identification of key premises is crucial to inform vector control operations—an activity which can be conducted through pupal/demographic surveys of household water containers.

However, the ubiquity of water containers tends to make pupal/demographic surveys laborious.^15^ Therefore, additional methods have been explored to identify key premises without needing to do extensive pupal/demographic surveys, or enter premises, because owners refusing access to premises has been reported as a key challenge.^16^ The Premise Condition Index (PCI) is one such approach that could help prioritize outbreak response in terms of *Aedes* infestation risk.^9^ This index evaluates the shade, house, and yard conditions of premises to produce risk strata. In addition to targeting treatment of key premises, this method could potentially be used to prioritize villages or other geographical areas when funding or human resources are insufficient to treat all outbreak areas.

Existing evidence of the value of the PCI to inform vector control programs is mixed. The PCI was first described and evaluated in Queensland, Australia, where it was found that inspecting 9.5% of premises with a high PCI score of 8–9 (out of 9) identified 54.4% of infested premises. Comparison of highest to lowest scores indicated a risk of infestation 5.6 times higher, with the number of positive containers 14.3 times higher.^9^ Other studies found a correlation between PCI and the number of positive containers^17–21^ and/or positive premises.^19,20,22^ Premise Condition Index has also been used to create risk strata, where a positive correlation (*r* = 0.968, *P* < 0.01) was identified in Brazil between risk strata and houses positive for *Aedes albopictus* eggs.^23^ By contrast, other studies found no association of *Aedes* mosquitoes with PCI.^24,25^ Furthermore, serious limitations or missing information exist in many of the past studies. Some studies report associations but do not provide data related to PCI in their article^18,21,24,26,27^ or relied on low sample sizes with wide CIs.^19^

Considering these uncertainties, this study aimed to assess whether higher mean densities of adult female *Aedes* mosquitoes and *Aedes* pupae are associated with worse premise conditions, as measured by PCI; and whether this association leads to reliable predictions of which premises should be targeted for interventions. The study was conducted in Cambodia, a country with one of the highest per capita incidence rates in Asia, at 0.7–3.0 per 1,000 population per year,^28,29^ and recurring outbreaks every 3–5 years.^30^ The Cambodia National Dengue Control Program developed a protocol to respond to outbreaks, defined as three or more cases in one village per year, which includes applying larvicides (e.g., temephos), adulticides (e.g., thermal fogging with pyrethroids), and distributing information, education, and communication materials. These activities are implemented throughout the entire outbreak villages and can require significant financial and human resources, especially if distances between villages and the number of outbreaks are large. In this setting, if shown to be effective, PCI could potentially be used to prioritize interventions when funds are insufficient to treat all houses or geographic areas. An advantage of the index is that it can be completed quickly and there is no need to enter houses. Although previously published evidence on the relevance of the PCI varies by geography and mosquito life stage, no studies and field evaluations have previously been reported from Cambodia or Southeast Asia.

## MATERIALS AND METHODS

### Study setting.

The data used in this study were collected during a cluster randomized trial on the effect of guppy fish and pyriproxyfen on entomological outcomes,^31^ conducted in 30 clusters in two operational districts within Kampong Cham province. Each cluster had an average of approximately 200 households or 1,000 individuals and included one or more villages that were separated by neighboring villages by at least 200 m. Kampong Cham has one of the highest dengue incidence rates in Cambodia (1.6 cases per 1,000 people per year) and the environmental characteristics are similar to most dengue-endemic areas of Cambodia (H. Ra, personal communication). The dry season lasts from December to April, the light rain season from April to July, and the heavy rain season from August to October. This study only uses data from the pre-intervention baseline surveys and control clusters, which did not receive an intervention, of the aforementioned trial and are considered to be more representative of the typical conditions in the area. More detailed information about the study site can be found in the trial protocol.^31^

### Outcomes.

The primary outcome was the association between PCI (defined below) and the mean density of adult female *A. aegypti* at household level. Secondary outcomes include the association between 1) PCI and the mean density of adult female *A. aegypti* at cluster level and 2) association between PCI and the number of *Aedes* pupae per household and per cluster.

### Mosquito collection and PCI scoring.

Data were collected at four time points covering all three main seasons: survey 1 was in October/November 2015 during the rainy season, survey 2 was in February/March 2016 during the dry season, survey 3 was in May/June 2016 during the light rain season, and survey 4 was in September/October 2016 in the heavy rain season. The survey methodology was developed following the WHO guidelines for entomological collections.^30^ The survey team consisted of experienced government staff who received 3 days training before the start of the surveys. Twenty surveyors completed each of the four entomology surveys within 20 days. All tools and materials were pretested during training. Houses within each cluster were selected using a random-number generator applied to the village list managed by the village head.

Larvae and pupae collection were completed using the five-sweep net method^15^ for containers larger than 50 L. For this method, a net of size 20 cm by 33 cm was used. Surveyors turned the net in an anti-clockwise manner five times, then waited 1 minute and performed one sweep from the bottom. This method can sample around 35% of larvae and 31% of pupae, and the total number estimated by an adjustment factor.^15^ For containers of less than 50 L, all the water was poured through the sweep net. All containers within selected households were inspected. All pupae and larvae were put in a plastic bag, labeled, and taken back to the provincial laboratory for identification to the species level for *Aedes*, otherwise to genus.

The adult resting catch was completed using a battery-powered, portable aspirator (Camtech, Phnom Penh, Cambodia) for 10 minutes per house in the bedrooms and living spaces, starting in the bedroom and aspirating up and down the wall (from floor to 1.5 m) around the home in a clockwise manner. The mosquitoes were kept in a screw-top container inside a cold box and transported to the provincial laboratory for identification to the species level for *Aedes*, otherwise to genus. All adult *Aedes* mosquitoes were sexed. After identification, all mosquitoes were taken to the United States Naval Medical Research Unit-2 in Phnom Penh where entomologists confirmed identification of a random sample of 50% of immature and adult mosquitoes. Each house in the survey was scored on the degree of shade, condition of house, and condition of yard according to the method developed by Tun-Lin et al.^9^ Each category is scored from 1 to 3, and the sum represents the PCI score. The teams were provided with objective measures for scoring in each category (see Table 1), a laminated sheet including pictures of example premises for each score, and given training to standardize scoring between the three teams. In addition, a fourth category representing the source of water was scored; however, because of the homogeneity of water infrastructure, the results are not reported here.

**Table 1:** Measures for scoring the Premise Condition Index

Premise variables	Description	Classification score
P1. House condition	a. Well-maintained, e.g., newly painted or new house	1
	b. Moderately well-maintained house	2
	c. Not well-maintained house, e.g., paint peeling, broken items visible, dilapidated old house	3
P2. Yard condition	a. Tidy yard, e.g., no rubbish or trash evident, well-maintained gardens, and lawn	1
	b. Moderately tidy yard	2
	c. Untidy yard, rubbish and trash abundant, and the garden or lawn with overgrown grass	3
P3. Shade condition	a. Very little or no shade (< 25%), e.g., no major trees or bush	1
	b. Some shade (> 25% but < 50%)	2
	c. Plenty of shade, > 50%, e.g., large trees evident, layers of shrubs, green house, plastic tarp sheet, or overhanging roofs used	3
P4. Water supply and storage	a. Piped water supply only	1
	b. Well water supply only	2
	c. Rainwater and/or river water	3

### Climate.

General climate data (rainfall, temperature, and humidity) were recorded at one of the intervention health centers using a rain gauge and a Hobo™ onset data logger (Onset Computer Corporation, Bourne, MA) (all villages included in the study have virtually the same climate). Data from the all United States National Aeronautics and Space Administration satellites on climate are also available to double check the accuracy of these measurements.

### Sample size.

Sample size was determined for the needs of the corresponding trial and is discussed in length in the protocol.^31^ However, the sample size is at least as large as four other studies which reported a significant association or correlation of PCI with houses or containers with *Aedes* mosquitoes.^19–21,32^

### Statistical analysis.

All analyses were performed in R Studio version 3.5.0 (Murray Hill, NJ) and Stata^®^ version 14.2 (College Station, TX). The association between *Aedes* density and PCI was assessed through negative binomial regression using the number of adults per household as the response and a logarithmic link function. Hence, this analysis yields density ratios as an outcome measure. Models combined data from all seasons and included survey as a fixed effect term. Additional models including an interaction term of survey and PCI were also run. A likelihood ratio test showed the interaction term to not be statistically significant (*P* = 0.07), and therefore, the model with interaction was not included in the results. A similar model was used for the secondary outcomes, with the numbers of pupae, rather than adults, as the response. Additional zero-inflated models were fitted; however, the model fit better without zero inflation. All models used the robust sandwich estimator of standard errors^33^ to account for correlation of responses within clusters.

Associations between PCI and vector density are necessary but not sufficient for PCI to have sufficient sensitivity and specificity to be efficient in practice. Receiver operating characteristic (ROC) curves were used to ascertain the ability of PCI to predict the premises in the top quartile of mosquito biomass. Their accuracy was classified according to the value of the area under the ROC curve (AUC): not informative (AUC ≤ 0.5), rather low accuracy (0.5 < AUC ≤ 0.7), accuracies useful for some purposes (0.7 < AUC ≤ 0.9), and rather high accuracy (0.9 < AUC).^34^

### Ethical approval.

Ethical clearance was received by the Cambodian National Ethics Committee for Health Research on October 9, 2014 (ethics reference number 0285). In addition, ethics approval was received from the London School of Hygiene and Tropical Medicine Observational/Interventions Research Ethics Committee (ethics reference number 8812).

## RESULTS

During the study period, a total of 2,400 premises were inspected for the presence of immature and adult *Aedes* and assigned PCI scores*.* The average monthly rainfall during the study was 11 mm during the dry season (December–April), 139 mm during the light rain season (May–July), and 276 during the heavy rain seasons (August–November). As reported in Table 1, the majority of premises (89%) were assigned a PCI score between 5 and 7, and only 3% and 0.4% were assigned a PCI score of 8 or 9, respectively. The median of each component variable of the PCI was 2, and all possible values (1, 2 and 3) were observed for each component. This suggests that the overall PCI was not being dominated by any single component.

### Distribution of adult female *Aedes* mosquitoes by PCI ranking.

Table 2 shows 26% of houses overall had some adult female *Aedes*, with an average of 0.56 each (SD 2.18, range 0–82). The percentage of positive houses and *Aedes* females per house increased during the light rain season to 58% and 1.88 (SD 4.81, range 0–82), respectively. The percentage of houses positive for *Aedes* females varied among overall PCI scores (17–33%) and among different seasons (17–58%). The average number of *Aedes* females per house also varied widely among overall PCI scores (0.21–0.73) and over seasons (0.24–1.88). The highest numbers of positive houses and average number of adult female *Aedes* was among premises with PCI scores of 6 and 7. Table 3 shows that 46% of premises and 15% of containers were positive for *Aedes* pupae and/or larvae with an average of seven pupae per house. The proportion of positive premises varied quite widely between PCI scores (22–51%) and between surveys (36–71%) with light rain (peak) season having by far the highest proportion of positive houses (71%). The percent of containers positive for larvae or pupae also varied among PCI scores (7–20%) and surveys (10–21%). Only 1% of premises received a PCI score of three and a few of those premises had extremely high numbers of *Aedes* pupae. The particular reason for the large number of pupae is that two premises had a large water container used for animal husbandry that were not often cleaned and held hundreds of pupae. Table 4 shows the results of the negative binomial regression models for adult female *Aedes* mosquitoes. The model including two dependent variables (PCI scores and survey) was found to fit best. The number of adult *Aedes* females was positively associated with PCI (rate ratio [RR] per point = 1.16, 95% CI: 1.02–1.31). A cluster-level model of adult *Aedes* females by cluster had a slightly higher RR, although wider CIs (RR = 1.54, 95% CI: 1.11–2.08).

**Table 2:** Adult female *Aedes* indicators by Premise Condition Index (PCI) ranking over different seasons

PCI score	Number of houses (%)	Houses with at least one *Aedes* female (%)	*Aedes* females	Mean *Aedes* females per house (SD, minimum–maximum)
All time points combined (*n* = 30 clusters)				
3	30 (1)	5 (17)	11	0.37 (1.03, 0–5)
4	138 (6)	32 (23)	50	0.36 (0.85, 0–6)
5	623 (26)	133 (21)	224	0.36 (0.93, 0–8)
6	1,178 (49)	329 (28)	791	0.67 (2.78, 0–82)
7	327 (14)	97 (30)	239	0.73 (2.16, 0–30)
8	71 (3)	15 (21)	22	0.31 (0.77, 0–4)
9	9 (0)	3 (33)	3	0.33 (0.50, 0–1)
Missing	24 (1)	4 (17)	5	0.21 (0.51, 0–2)
Total	2,400 (100)	618 (26)	1,345	0.56 (2.18, 0–82)
October 2015 (heavy rain season): control at baseline (*n* = 10 clusters)				
3	3 (1)	0 (0)	0	0.00 (0, 0–0)
4	31 (8)	8 (26)	10	0.32 (0.60, 0–2)
5	126 (32)	23 (18)	38	0.30 (0.79, 0–5)
6	183 (46)	25 (14)	33	0.18 (0.52, 0–3)
7	41 (10)	11 (27)	19	0.46 (1.07, 0–6)
8	11 (3)	1 (9)	1	0.09 (0.30, 0–1)
9	0 (0)	0 (0)	0	–
Missing	5 (1)	0 (0)	0	0.00 (0, 0–0)
Total	400 (0)	68 (17)	101	0.25 (0.69, 0–6)
February 2016 (dry season) (*n* = 10 clusters)				
3	3 (1)	1 (33)	5	1.67 (2.89, 0–5)
4	14 (4)	4 (29)	7	0.50 (1.09, 0–4)
5	187 (47)	42 (22)	71	0.38 (1.03, 0–8)
6	161 (40)	47 (29)	106	0.66 (1.62, 0–14)
7	23 (6)	6 (26)	7	0.30 (0.56, 0–2)
8	6 (2)	1 (17)	1	0.17 (0.41, 0–1)
9	3 (1)	0 (0)	0	0.00 (0, 0–0)
Missing	3 (1)	0 (0)	0	0.00 (0, 0–0)
Total	400 (100)	101 (25)	197	0.49 (1.29, 0–14)
June 2016 (light rain season) (*n* = 10 clusters)				
3	4 (1)	1 (25)	1	0.25 (0.50, 0–1)
4	32 (8)	10 (31)	22	0.69 (1.35, 0–6)
5	54 (14)	29 (54)	64	1.19 (1.63, 0–7)
6	230 (58)	148 (64)	505	2.20 (5.8, 0–82)
7	78 (20)	42 (54)	160	2.05 (3.93, 0–30)
8	2 (1)	0 (0)	0	0.00 (0, 0–0)
9	0 (0)	0 (0)	0	–
Missing	0 (0)	0 (0)	0	–
Total	400 (100)	230 (58)	752	1.88 (4.81, 0–82)
October 2016 (heavy rain season) (*n* = 10 clusters)				
3	4 (1)	1 (25)	1	0.25 (0.5, 0–1)
4	13 (3)	2 (15)	2	0.15 (0.38, 0–1)
5	42 (11)	11 (26)	14	0.33 (0.61, 0–2)
6	280 (70)	50 (18)	68	0.24 (0.59, 0–3)
7	56 (14)	7 (13)	9	0.16 (0.46, 0–2)
8	4 (1)	2 (50)	2	0.50 (0.58, 0–1)
9	1 (0)	0 (0)	0	0.00 (0, 0–0)
Missing	0 (0)	0 (0)	0	–
Total	400 (100)	73 (18)	96	0.24 (0.57, 0–3)

**Table 3:** Immature *Aedes* indicators by Premise Condition Index (PCI) ranking over different seasons

PCI score	Number of houses (%)	Houses positive for *Aedes* larvae or pupae (%)	Number of containers (%)	Number of containers positive	Number of *Aedes* pupae	Mean pupae per house (SD, minimum–maximum)
All time points combined (*n* = 30 clusters)						
3	30 (1)	12 (40)	179 (1)	32	991	33 (145, 0–791)
4	138 (6)	59 (43)	723 (5)	109	887	6 (22, 0–166)
5	623 (26)	250 (40)	3,548 (26)	431	5,739	9 (105, 0–2,580)
6	1,178 (49)	578 (49)	7,016 (52)	1,060	8,588	7 (27, 0–585)
7	327 (14)	167 (51)	1,610 (12)	283	1,450	4 (12, 0–97)
8	71 (3)	35 (49)	283 (2)	56	286	4 (13, 0–81)
9	9 (0)	2 (22)	46 (0)	3	9	1 (2, 0–5)
Missing	24 (1)	11 (46)	124 (1)	18	49	2 (5, 0–16)
Total	2,400 (100)	1,102 (46)	13,529 (100)	1,992	17,999	7 (60, 0–2,580)
October 2015 (heavy rain season): control at baseline (*n* = 10 clusters)						
3	3 (1)	1 (33)	18 (1)	2	11	4 (6, 0–11)
4	31 (8)	12 (39)	117 (8)	23	92	3 (9.6, 0–50)
5	126 (32)	51 (40)	483 (31)	78	594	5 (15, 0–129)
6	183 (46)	72 (39)	726 (47)	105	759	4 (11, 0–82)
7	41 (10)	18 (44)	142 (9)	28	205	5 (12, 0–59)
8	11 (3)	3 (27)	33 (2)	7	3	0 (1, 0–3)
9	0 (0)	0 (0)	0 (0)	0	0	–
Missing	5 (1)	4 (80)	25 (2)	4	12	2 (5, 0–12)
Total	400 (100)	161 (40)	1,544 (100)	247	1,676	4 (12, 0–129)
February 2016 (dry season) (*n* = 10 clusters)						
3	3 (1)	1 (33)	35 (1)	10	124	41 (72, 0–124)
4	14 (4)	6 (43)	169 (5)	10	98	7 (24, 0–91)
5	187 (47)	59 (32)	1,517 (42)	136	653	3 (10, 0–89)
6	161 (40)	62 (39)	1,584 (44)	167	947	6 (19, 0–131)
7	23 (6)	10 (43)	224 (6)	17	81	4 (8, 0–26)
8	6 (2)	3 (50)	46 (1)	8	18	3 (7, 0–18)
9	3 (1)	0 (0)	22 (1)	0	0	0 (0, 0–0)
Missing	3 (1)	1 (33)	23 (1)	2	0	0 (0, 0–0)
Total	400 (100)	142 (36)	3,620 (100)	350	1,921	5 (16, 0–131)
June 2016 (light rain season) (*n* = 10 clusters)						
3	4 (1)	2 (50)	16 (1)	5	6	2 (3, 0–6)
4	32 (8)	20 (63)	152 (6)	33	272	9 (22, 0–121)
5	54 (14)	33 (61)	364 (15)	53	607	11 (29, 0–148)
6	230 (58)	174 (76)	1,480 (61)	342	2,741	12 (35, 0–330)
7	78 (20)	52 (67)	411 (17)	86	296	4 (11, 0–71)
8	2 (1)	1 (50)	5 (0)	1	0	0 (0, 0–0)
9	0 (0)	0 (0)	0 (0)	0	0	–
Missing	0 (0)	0 (0)	0 (0)	0	0	–
Total	400 (100)	282 (71)	2,428 (100)	520	3,922	10 (30, 0–330)
October 2016 (heavy rain season) (*n* = 10 clusters)						
3	4 (1)	1 (25)	40 (2)	2	32	8 (16, 0–32)
4	13 (3)	7 (54)	99 (4)	15	115	9 (26, 0–92)
5	42 (11)	19 (45)	250 (10)	33	180	4 (15, 0–91)
6	280 (70)	96 (34)	1,698 (70)	146	807	3 (9, 0–96)
7	56 (14)	20 (36)	312 (13)	30	98	2 (5, 0–25)
8	4 (1)	4 (100)	26 (1)	7	13	3 (4, 0–9)
9	1 (0)	0 (0)	4 (0)	0	0	0 (0, 0–0)
Missing	0 (0)	0 (0)	0 (0)	0	0	–
Total	400 (100)	147 (37)	2,429 (100)	233	1,245	3 (10, 0–96)

**Table 4:** Association between each Premise Condition Index point and the mean density of *Aedes* adult females and pupae at household and cluster level over multiple seasons

	Adult *Aedes*	*Aedes* pupae
By household		
Unadjusted	1.25 (1.11–1.39), *P ≤* 0.01	0.74 (0.57–0.96), *P* = 0.02
Adjusted for survey	1.16 (1.02–1.31), *P* = 0.02	0.74 (0.59–0.93), *P* = 0.01
By cluster		
Unadjusted	1.80 (1.12–2.88), *P* = 0.01	0.79 (0.32–1.93), *P* = 0.60
Adjusted for survey	1.52 (1.11–2.08), *P* = 0.01	0.78 (0.35–1.73), *P* = 0.55

### Correlation of immature *Aedes* mosquitoes with PCI.

Table 4 also shows the results of negative binomial regression models for *Aedes* adults and pupae. At the house level, the number of pupae were statistically significantly negatively associated with PCI scores (RR = 0.74, 95% CI: 0.59–0.93), whereas the number of adult A*edes* were significantly positively associated with PCI scores (RR = 1.16, 95% CI: 1.02–1.31). The model investigating the correlation between number of *Aedes* pupae and PCI was not significant at the cluster level, whereas the model for adults was positively associated (RR = 1.52, 95% CI: 1.11–2.08).

### Receiver operating characteristic curve analysis for predicting the top quartile of adult *Aedes* mosquitoes.

**Figure 1.**
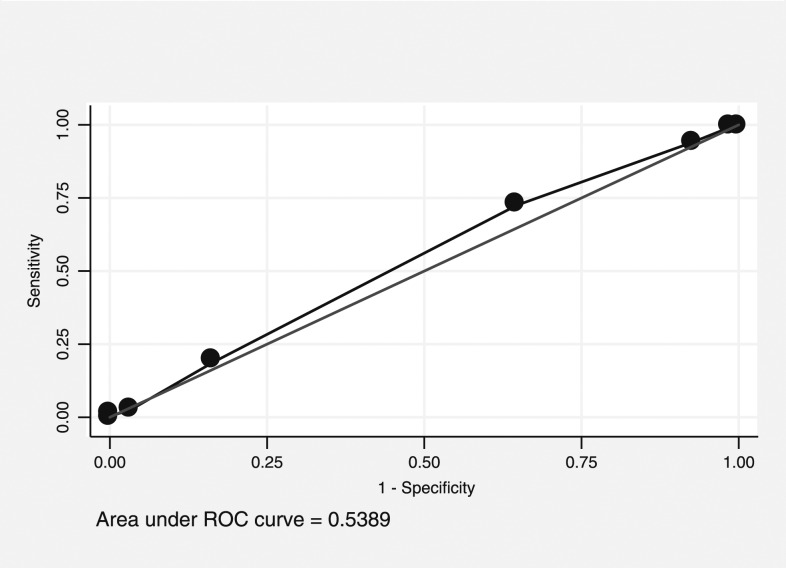
Receiver operating characteristic (ROC) curve of Premise Condition Index and prediction values in predicting the premises with the top quartile of adult mosquito density.

**Figure 2.**
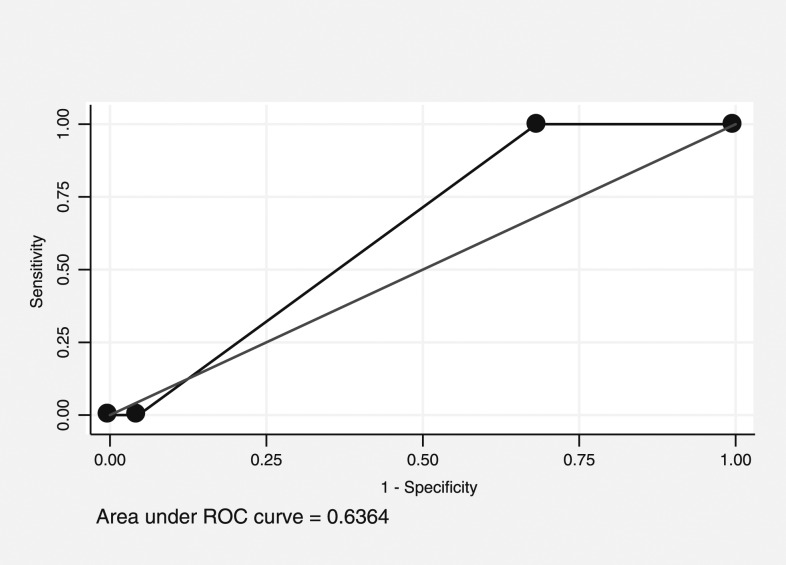
Receiver operating characteristic (ROC) curve of Premise Condition Index and prediction values in predicting the clusters with the top quartile of adult mosquito density.

The PCI score was considered to have “rather low accuracy” predicting premises in the top quartile of adult female *Aedes* mosquitoes, with an AUC of 0.54 (95% CI: 0.52–0.56, Figure 1). A cut point of 5 had high sensitivity (94%) and low specificity (7%), whereas 7 had low sensitivity (19%) and high specificity (83%). For clusters, the PCI score was also considered to have “rather low accuracy,” with an AUC of 0.64 (95% CI: 0.44–0.80, Figure 2). No cut point for either curve gives an adequate combination of sensitivity and specificity.

### Receiver operating characteristic curve analysis for predicting the top quartile of *Aedes* pupae.

**Figure 3.**
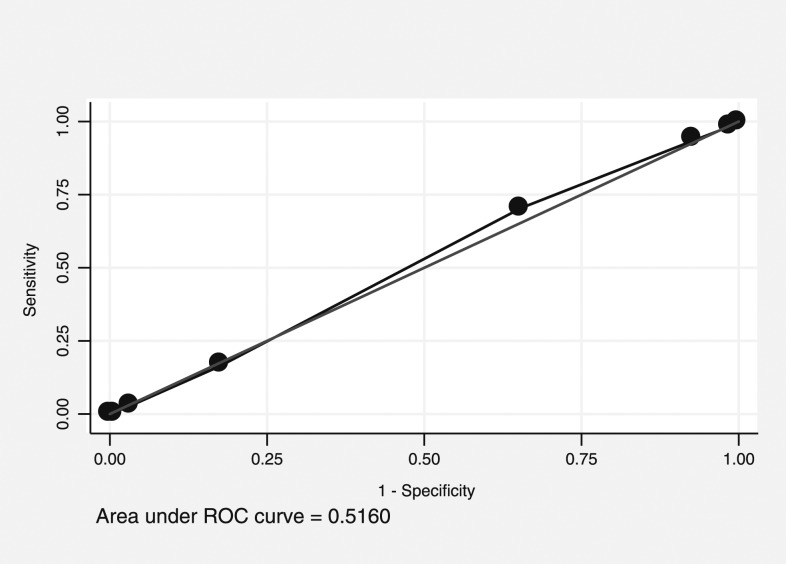
Receiver operating characteristic (ROC) curve of Premise Condition Index and prediction values in predicting the premises with the top quartile of immature mosquito density.

**Figure 4.**
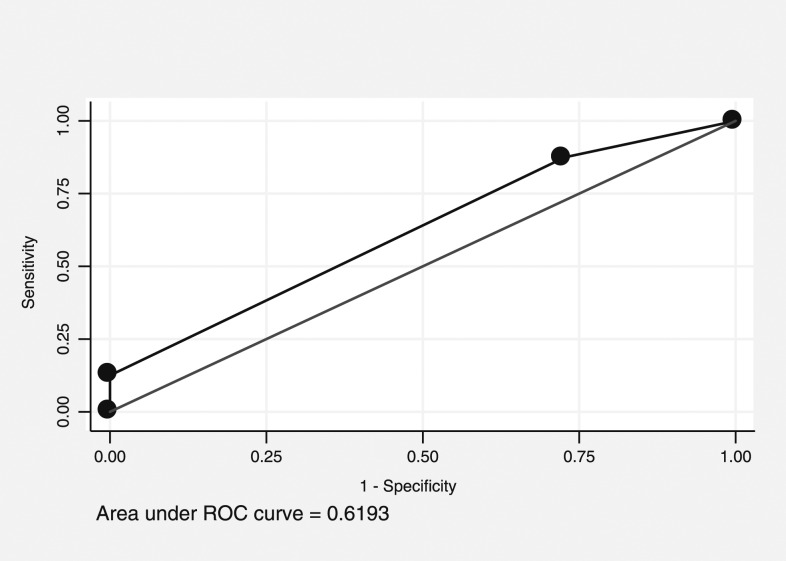
Receiver operating characteristic (ROC) curve of Premise Condition Index and prediction values in predicting the clusters with the top quartile of immature mosquito density.

The PCI score was considered to have “rather low accuracy” when predicting premises in the top quartile for *Aedes* pupae, with an AUC of 0.52 (95% CI: 0.50–0.54, Figure 3). A cut point of 5 had high sensitivity (93%) and low specificity (7%), whereas 7 had low sensitivity (16%) and high specificity (83%). For clusters, the PCI score was again considered to have “rather low accuracy” when predicting the clusters in the top quartile for *Aedes* pupae, with an AUC of 0.62 (95% CI: 0.44–0.80, Figure 4). No cut point for either curve gives an adequate combination of sensitivity and specificity. This low degree of accuracy is consistent with the negative association presented earlier.

## DISCUSSION

The PCI was found to be weakly associated with the density of adult female *Aedes* at the household and cluster level, and negatively associated with the number of *Aedes* pupae at the household level*.* Therefore, our hypothesis that higher mean densities of *Aedes* would be associated with worse premise conditions was correct for adult females, but not for pupae. The five premises with the highest number of *Aedes* pupae represented 25% of the total pupae and had relatively lower PCI scores (one house had a PCI of 3). This may have been because the most productive containers were large water storage containers for animal husbandry that are not frequently changed or replaced. More wealthy families and those with nicer houses may be more likely to have lots of farm animals and, therefore, need these large water storage containers. In contrast, 30 premises (2.5%) with the most adult female *Aedes* mosquitoes represented 25% of adult females and they tended to have relatively higher PCI scores (none had scores below 5). Therefore, the relative impact of one or two households has less weight on the overall measure with the adults than with immatures. Similar results have been found in other studies and resulted in affirmations of PCI’s effectiveness and suggestions on how to incorporate it into national control programs. Similar positive associations in Mexico (odds ratio [OR] = 1.27, *P* = 0.001) between PCI and *Aedes* larvae resulted in researchers concluding that the PCI can be an adequate estimator of the *Ae. Agypti* infestation rate.^22^ In Brazil, researchers found a positive correlation between PCI and houses positive for *Aedes* eggs (*r* = 0.97, *P* < 0.01) and stated that the results clearly showed the usefulness of the method.^23^ They went one step further and suggested “in the case of dengue outbreaks, by having all representative house indices of the region, it will be much easier and less expensive to control the epidemic.” Positive correlations between PCI and house positivity for larvae, pupae, and adult *A. aegypti* (*P* > 0.05) led authors to advocate to the Brazilian Dengue Control Program the use of PCI to schedule the vector control teams’ visits with different frequencies based on PCI scores.^17^ In Mexico, a significant positive correlation between average PCI of a location and the house index was found (OR = 1.37, *P* = 0.007), and it was noted that in the near future, the authors expected to use information derived from PCI to “focalize integrated dengue vector control on houses/city blocks/neighborhoods/areas with high levels of PCI (6–9).”^20^ These examples show how relatively weak evidence has been used to advocate for PCI’s use and integration into national policy.

However, finding statistically significant correlations does not always mean that the variables will be good predictors.^35^ In our study, ROC curves showed that PCI had “rather low accuracy” (AUC = 0.54 and 0.52, respectively) to predict premises in the top quartile for *Aedes* adult females and pupae. Additional ROC curves measuring the ability of PCI to predict clusters (as opposed to houses), which represent the top quartile of *Aedes* adult females and pupae also found it to have “rather low accuracy” (AUC = 0.64 and 0.62, respectively). This is especially true when using highly variable outcomes such as immature measures. Therefore, control programs may want to use care when interpreting PCI associations in their area.

There are also several limitations of the PCI methodology to consider including that nonresidential premises, vacant lots, and construction worksites are often not ranked. Andrighetti et al.^17^ noted that 21% of the premises in their study could not be ranked and harbored 11.6% of larvae, 20.9% of pupae, and 20.8% of adults. In our study, we did not include vacant lots, schools, monasteries, or other public areas and, therefore, results may not be representative of those areas. In addition, the inability of the inspector to inspect or see into rear yards in some study settings may lead to misclassification.^19^ One of the key weaknesses that has been widely reported is that the scoring may not be standardized across individuals, teams, or organizations.^19^ One potential way to reduce this variability would be to use drones to take aerial photographs that could be scored by one individual or team. Another way could be to use PCI to classify geographical locations where it has been shown useful would be to assign one team to categorize the areas in known hot spots in advance of outbreaks. Then, the scores could be used to try to identify which hot spots or villages to target when resources are scarce. Nevertheless, it is unknown how use of PCI to prioritize households or geographical areas would be accepted within the communities.^25^ In addition, this would only work if PCI was not variable between seasons and years.

These results may not be generalizable to areas with more variability in housing conditions, different ecological conditions, or different mosquito abundance profiles. Considerable resources need to be invested in ensuring teams have standardized scoring of PCI, the corresponding PCI cutoffs are followed correctly, and evaluating the acceptance of individuals or communities who are not prioritized. These resources may be better spent evaluating other methods to target premises or spent generally on *Aedes* control. Future studies could evaluate the use of PCI in other geographical settings, the effectiveness of PCI to identify premises with dengue infection, or the acceptance by the community of PCI’s use where it is found to be effective.
